# Genome-wide identification and analysis of long non-coding RNAs involved in fatty acid biosynthesis in young soybean pods

**DOI:** 10.1038/s41598-021-87048-7

**Published:** 2021-04-07

**Authors:** Bohan Ma, Aijing Zhang, Qiuzhu Zhao, Zeyuan Li, Abraham Lamboro, Haobo He, Yue Li, Suqi Jiao, Shuyan Guan, Siyan Liu, Dan Yao, Jun Zhang

**Affiliations:** 1 College of Life Sciences, Jilin Agricultural University, Changchun, 130118 China; 2 College of Agronomy, Jilin Agricultural University, Changchun, 130118 Jilin China

**Keywords:** Molecular biology, Plant sciences

## Abstract

Long non-coding RNAs (lncRNAs) are non-coding RNAs of more than 200 nucleotides. To date, the roles of lncRNAs in soybean fatty acid synthesis have not been fully studied. Here, the low-linolenic acid mutant ‘MT72′ and the wild-type control ‘JN18′ were used as materials. The lncRNAs in young pods at 30 and 40 days (d) after flowering were systematically identified and analyzed using transcriptome sequencing technology combined with bioinformatics tools. A total of 39,324 lncRNAs and 561 differentially expressed lncRNAs were identified. A lncRNAs-miRNAs-protein-coding genes (mRNAs) network was constructed, and 46 lncRNAs, 46 miRNAs and 137 mRNAs were found to be correlated. Gene Ontology (GO) and Kyoto Encyclopedia of Genes and Genomes (KEGG) pathway analysis of 12 targeted mRNAs in the competing endogenous RNA network showed that these lncRNAs may be involved in the biological processes of fatty acid transport, lipid synthesis and cell division. Finally, the expression levels of differentially expressed lncRNAs, miRNAs and mRNAs were verified using qRT-PCR. The expression patterns of most genes were consistent with the sequencing results. In conclusion, new information was provided for the study of fatty acid synthesis by lncRNAs in young soybean pods.

## Introduction

Soybean [*Glycine max* (L.) Merr] is an important oil crop that is planted worldwide^1^. Soybean oil is the world’s vegetable oil. Its synthetic pathway can be divided into the de novo synthesis of fatty acids, the synthesis of triacylglycerol and the formation of oils and fats. Soybean oil is synthesized mainly in plastids and the endoplasmic reticulum. Fatty acid precursors produce saturated fatty acids 18:1-acyl-acyl carrier protein (ACP) by polymerization. The production of 18:3-ACP is catalyzed by stearyl-ACP desaturation enzyme. Then, using 18:1-ACP as a substrate of free fatty acids, acylcoenzyme A synthase synthesizes acyl coenzyme A, which enters the endoplasmic reticulum. Soybean oil is then synthesized by a series of enzymes. Fatty acids account for more than 90% of soybean oils fat content, and the content and composition of the fatty acids are important indexes to determine the oil’s quality. Saturated and unsaturated fatty acids are made up of fatty acids^2^. Oleic, linoleic and linolenic acids are unsaturated fatty acids. Unsaturated fatty acids have important functions that lengthen plant cell life, reduce the cholesterol content in the human body and reduce brain thrombosis formation. The human body cannot synthesize linoleic and linolenic acid. Therefore, they must be obtained from food. Thus, breeding new soybean varieties with high monounsaturated fatty acid contents and low contents of polyunsaturated fatty acid contents has become an urgent task for breeders.

Since the rise of miRNA research in 2005, non-coding RNAs (ncRNAs) have been at the forefront of epigenetic research^3^. After miRNAs, ncRNAs of different types and sources, such as long non-coding (lncRNAs) and circular RNAs, were successively discovered^4^, and their functions are being continuously revealed. NcRNAs play important regulatory roles in various physiological processes of the body. The study of ncRNAs has gradually shifted from focusing on the function of a single RNA to the multi-factor and even omics-based coordinated regulation of multiple RNAs. LncRNAs are a class of ncRNAs that are longer than 200 nt, with a promoter substructure and polyA tail, and both tissue and space–time specificity^5^. LncRNAs expression levels are different in different plant tissues. NcRNAs were originally thought to be transcriptional ‘noise’ because of their low expression levels and sequence conservation. However, recent studies have found that lncRNAs are key regulators of cellular processes and can play roles at the transcription level as *cis*- or *trans*-regulators of gene expression with functions in regulating plant growth and development^6^. Before 2000, there were no research reports from China related to lncRNAs, but by 2017, more than 3,500 papers had been published. By 2019, there have been nearly 7,000 published papers on lncRNAs in plants. Those in maize (*Zea mays* L.), Arabidopsis (*Arabidopsis thaliana* L.), rice (*Oryza sativa* L.) and other oil crops accounted for more than 50% of the research. To date, a large number of lncRNAs have been identified in soybean (*Glycine max* L.)^7^, Arabidopsis (*Arabidopsis thaliana* L.)^8^, wheat (*Triticum aestivum* L.)^9^, rice (*Oryza sativa* L.)^10^, maize (*Zea mays* L.)^11^, tomato (*Solanum lycopersicum* L.)^12^ Brassica napus^13^. Chen identified 3,030 long intergenic non-coding RNAs (lincRNAs) and 275 natural antisense transcripts (lncNATs) in soybean roots by high-throughput sequencing^14^. And these lncRNAs induced by continuous salt-stress and their potential functions in soybean roots were explored. Lin comprehensively identified lncRNAs from different soybean tissues under different conditions^15^. In addition, publicly available soybean transcriptome data including 322 samples were analyzed totally, and 69,000 lncRNA gene loci were identified. A previously unreported subset of small peptide-coding transcripts was identified from these lncRNA loci via tandem mass spectrometry. Golicz found 6,018 lincRNAs sites through RNA-seq technology in 37 samples from 9 different tissues of soybean^16^. The co-expression analysis of lncRNAs and protein-coding genes showed that lncRNAs may be involved in stress response, signal transduction and development process. Kang systematically analyzed lncRNAs in the shattering-sensitive (SS) and shattering-resistant (SR) of soybean pods by RNA-Seq^17^, and 225 differentially expressed lncRNAs were identified. Finally, the potential genes and molecular pathways of differences in soybean pod dehiscence were further explored through the co-expression network of lncRNAs and protein-coding genes. At present, the research on the function of lncRNAs is one of the hotspots and difficulties in biological research. The identification and analysis of lncRNAs in soybean will be helpful to understand the characteristics and related biological functions of lncRNAs in other oil-bearing crops better.

LncRNAs cannot encode proteins but are structurally similar to known protein-coding genes. Some plant lncRNAs can act as competing endogenous RNAs (ceRNAs) and regulate miRNAs through targeting. Therefore, the interaction between a miRNA and its targets may be blocked to affect plant growth and development. For example, Bardou reported that lncRNAs Induced by Phosphate Star-Vation1 (IPS1) induced by low phosphorus in A. thaliana removes the inhibition of miR399 on target gene PHO2 by simulating the target gene^18^. Therefore, the phosphorus reaction process is regulated to reach a stable state. Wang identified several lncRNAs as target simulators of tomato miRNAs in response to tomato yellow leaf curl virus infection^19^. Recently, studies in B. napus predicted that 13 lncRNAs were precursors of 96 miRNAs. These miRNAs are involved in the infection of B. napus. Although there have been reports on the mechanism and functions of lncRNAs, their roles in plants are not fully understood. Therefore, the characteristics of different types of lncRNAs in plants need to be further studied to clarify their mechanism of action.

To date, a large number of lncRNAs regulating fat metabolism have been screened in human, mouse, pig, bovine and other species. Studies have indicated that lncRNAs are key regulators of human thermogenic adipocytes, and have revealed the role of lncRNAs in organelle communication and human energy metabolism^20^. LncRNAs have been identified as having potential functions in many plants. Yin reported that lncRNAs and related target genes in peony seeds may be involved in fatty acid synthesis and lipid metabolism^21^. Shen found that some lncRNAs in B. napus may play roles in lipid synthesis^22^. However, there were no research findings on the involvement of lncRNAs in soybean oil synthesis. In this study, the expression profiles of lncRNAs at different stages in the young pods of the low-linolenic acid mutant ‘MT72′ and wild-type ‘JN18′ were systematically identified and analyzed. Then, we compared the levels of differentially expressed lncRNAs with those of protein-coding genes, and finally, we inferred that some lncRNAs had potential functions related to the regulation of soybean lipid anabolism using a ceRNA co-expression network. This research provides insights into the regulation of ceRNAs and other biological processes in soybean, and it also provides theoretical basis into the functions of lncRNAs in soybean and other oil-bearing crops.

## Results

### Preliminary experiments of sequencing

**Figure 1:**
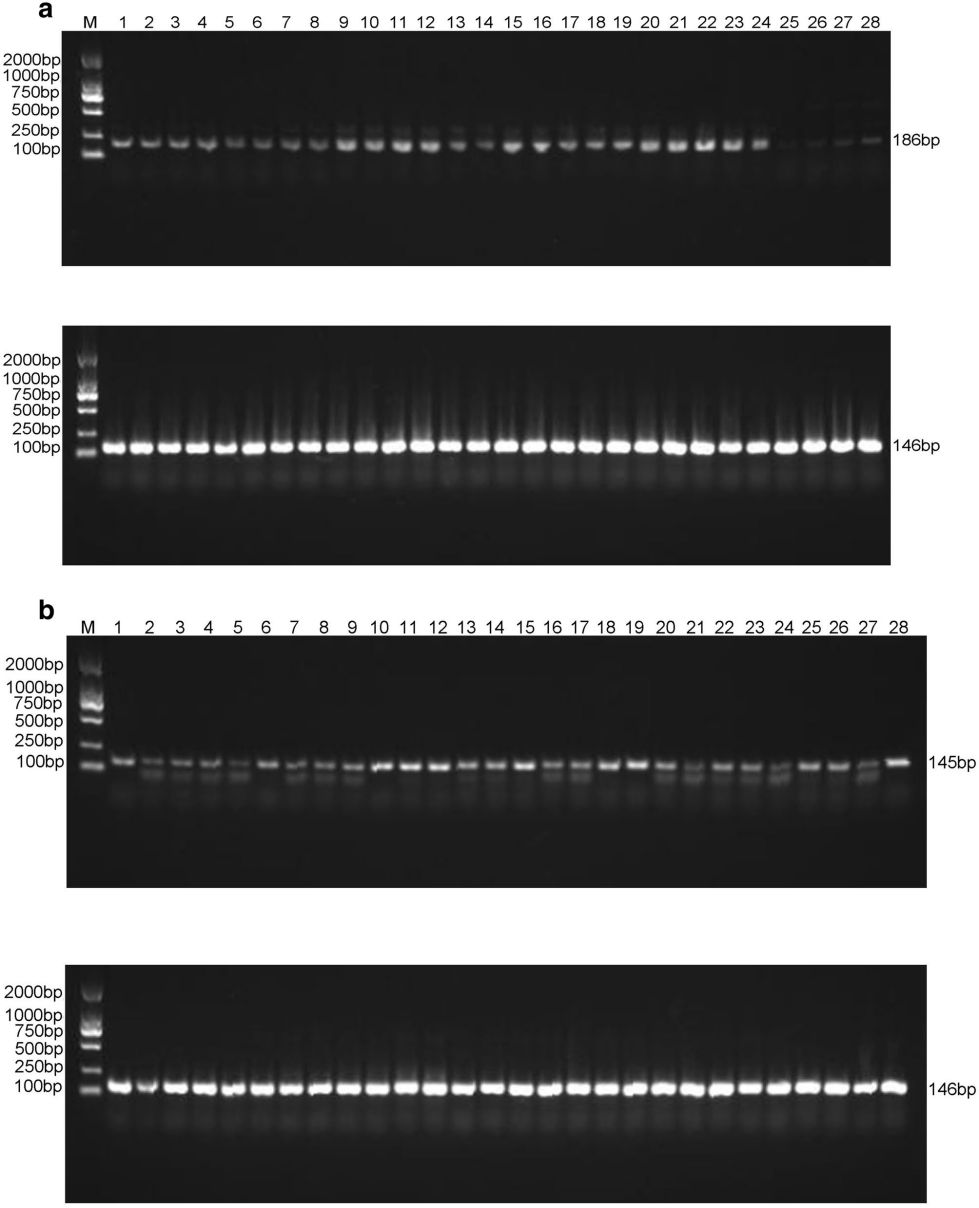

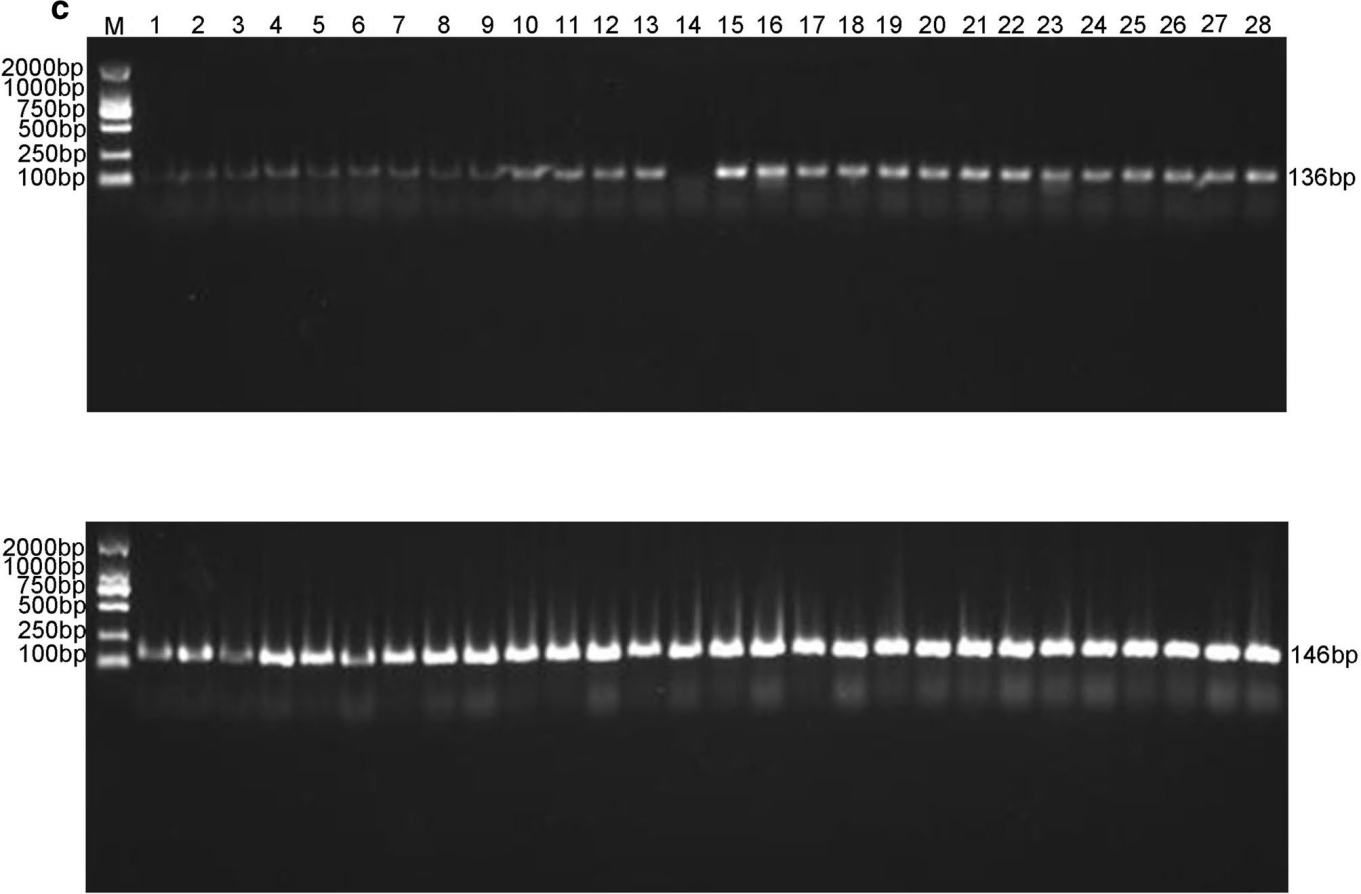
RT-PCR of *FAD3C-1*, *ACC*, *GAT* expression in seven developmental periods. (**a**) *FAD3C-1*, (**b**) *ACC*, (**c**) *GAT*. below the target gene is the internal control gene *Lectin*. Lane numbers indicate the soybean developmental stage as follows: lanes 1–4, 20 d after flowering, 5–8, 30 d after flowering, 9–12, 40 d after flowering, 13–16, 50 d after flowering, 17–20, 60 d after flowering, 21–24, 70 d after flowering, 25–28, 80 d after flowering.

**Figure 2:**
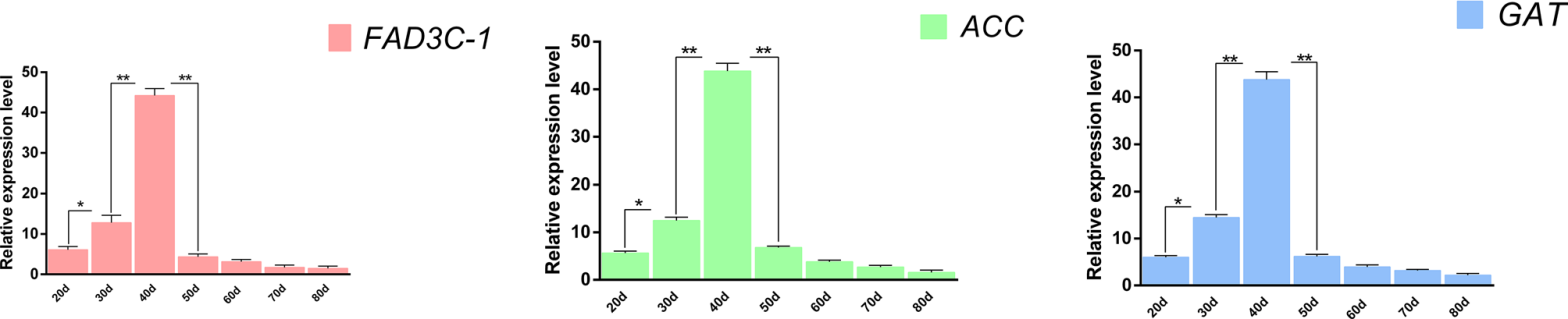
Analysis of relative expression levels of *FAD3C-1*, *ACC* and *GAT* genes in soybean during seven developmental stages from 20 to 80 d after soybean flowering. *Indicates a statistical difference at *p* < 0.05, **indicates a statistical difference at *p* < 0.01.

**Figure 3:**
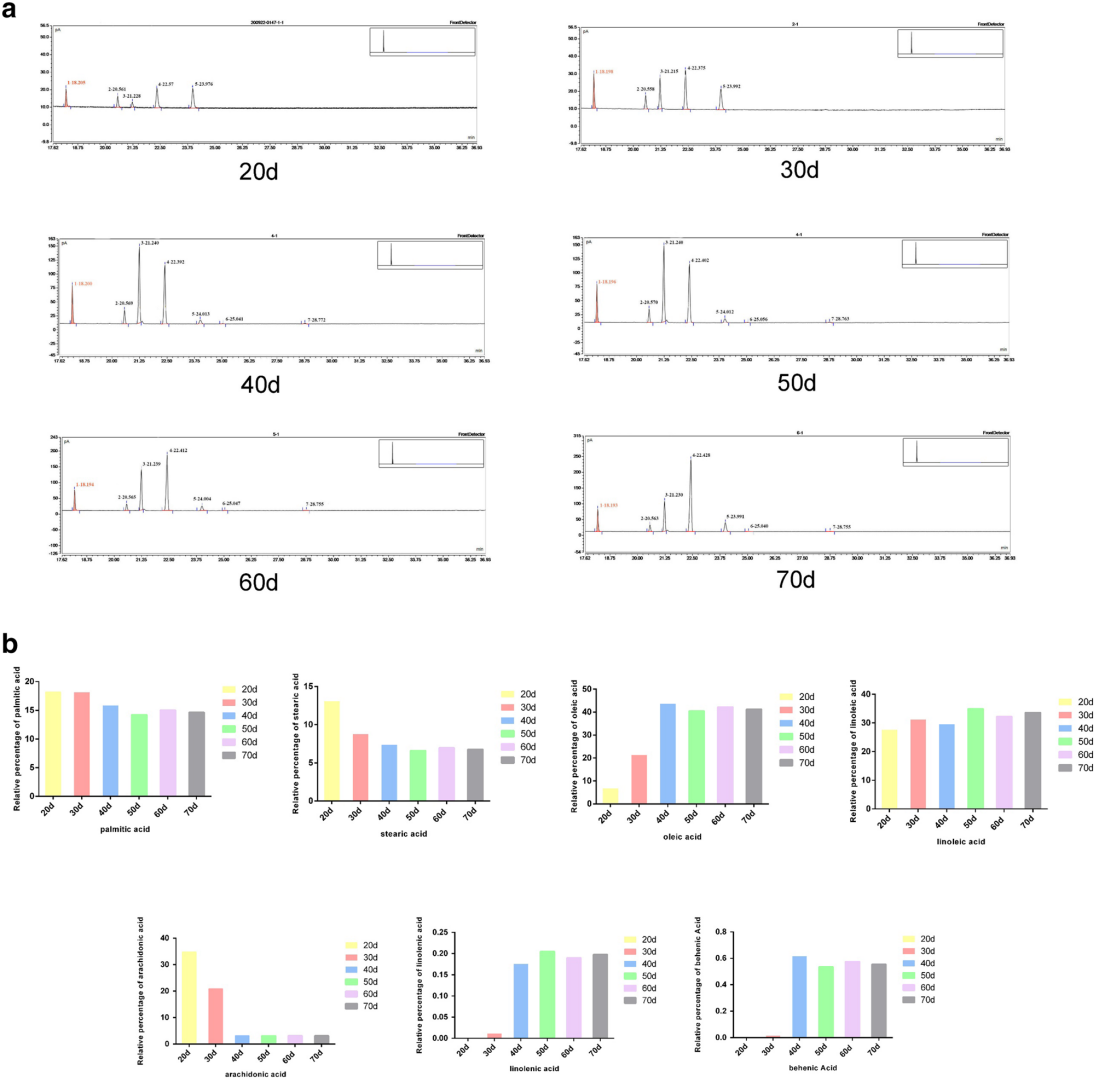
The gas chromatogram of soybean pods at different stages. (**a**) Gas chromatographic peak of fatty acid content in soybean at different stages (The starting points from left to right are palmitic acid, stearic acid, oleic acid, linoleic acid, arachidonic acid, linolenic acid and behenic acid). (**b**) The relative content of fatty acids in soybean in different periods.

The key enzyme-encoding genes *FAD3C-1*, *ACC*, and *GAT* in the soybean fatty acid synthesis pathway were selected as the target genes, and *Lectin* was the internal control gene. The soybean transcription level was analyzed in the corresponding period using RT-PCR and real-time fluorescence quantitative PCR (Fig. 1), and the correlations between the expression levels of key enzyme genes for oil synthesis and different developmental stages of soybean grains were determined. Additionally, changes in the oil accumulation levels of soybean ‘MT72′ and ‘JN18′ during different developmental periods were systematically analyzed. The results showed that the changes mostly occurred between 30 and 40 d after the flowering, and then the oil accumulation gradually decreased (Fig. 2). In order to further verify this result, gas chromatography experiments were carried out on fatty acids of soybean ‘JN18′ at different stages (Fig. 3).

### Identification and characterization of lncRNAs in soybean

**Figure 4:**
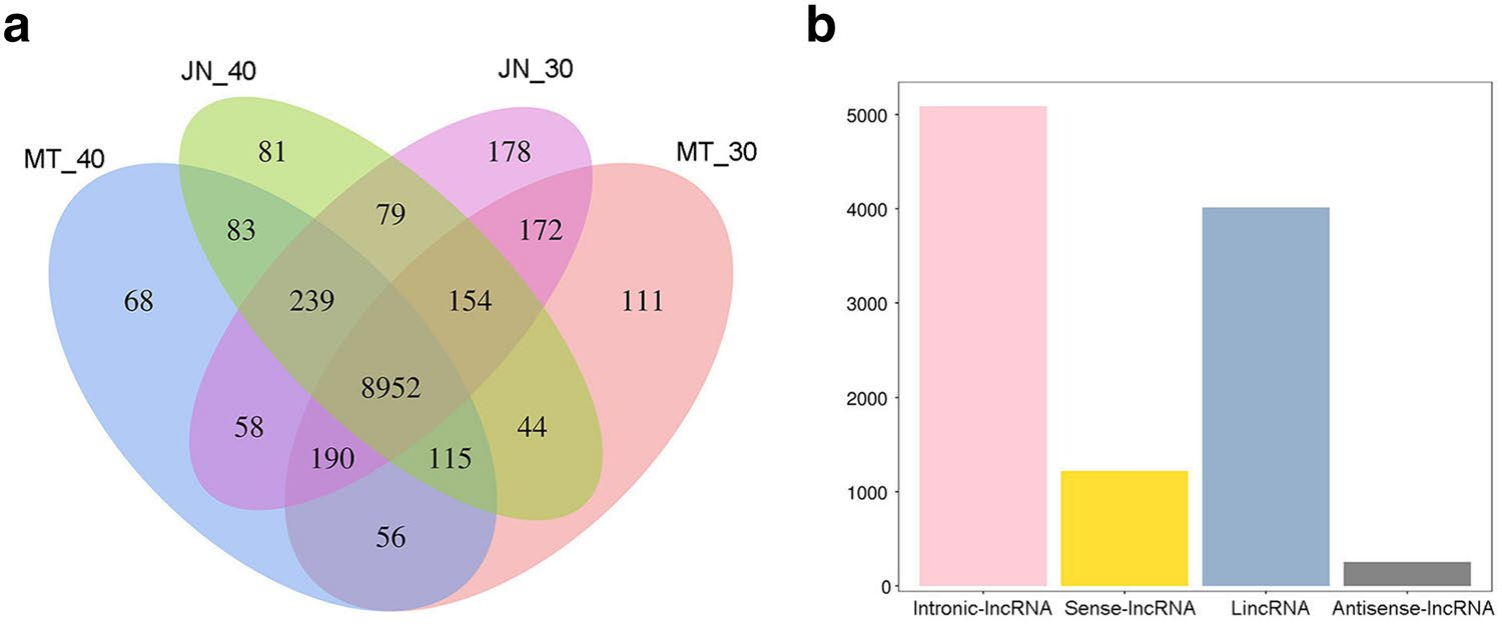
LncRNAs detected in MT72 and JN18 at 30 d and 40 d after soybean flowering. (**a**) Venn diagram of lncRNAs detected in each sample (MT_40, MT72 at 40 d after flowering, MT_30, MT72 at 30 d, JN_40, JN18 at 40 d, JN_30, JN18 at 30 d). (**b**) Chart showing the different types of lncRNAs.

In this study, two soybean cultivars were investigated by transcriptome sequencing at two seed development stages (30 and 40 d after flowering), with three biological replicates. The sequencing produced 300 million raw reads. After using Cutadapt^23^ to filter out the unqualified sequences, the clean data were obtained. More than 96% of the raw reads were clean reads (Supplementary Table 1). Clean Data were located by comparison with two soybean reference genomes, and reads were assembled and annotated using StringTie^24^, the latest transcription assembly software. In total, 52,507, 53,733, 53,601, 54,012, 52,085, 49,641, 51,779, 52,224, 52,926, 52,105, 52,070, and 51,551 mRNAs were identified from 12 cDNA libraries, including ‘MT72′ at 30 and 40 d after flowering (libraries MT30 and MT40, respectively) and ‘JN18′ at 30 and 40 d after flowering (libraries JN30 and JN40, respectively) (Supplementary Table 2). Then, the known mRNAs and transcripts less than 200 bp were removed, and the remaining transcripts were used to predict lncRNAs. The transcripts with protein-encoding potential were removed, and 9,761, 9,747, 9,794 and 10,022 lncRNAs were detected in MT40, JN40, MT30 and JN30, respectively. In this study, 39,324 lncRNAs were identified (Supplementary Table 3). The number of lncRNAs found in the two varieties at 30 d was higher than that detected at 40 d. There were 8,952 common lncRNAs in the four samples (Fig. 4a).

**Figure 5:**
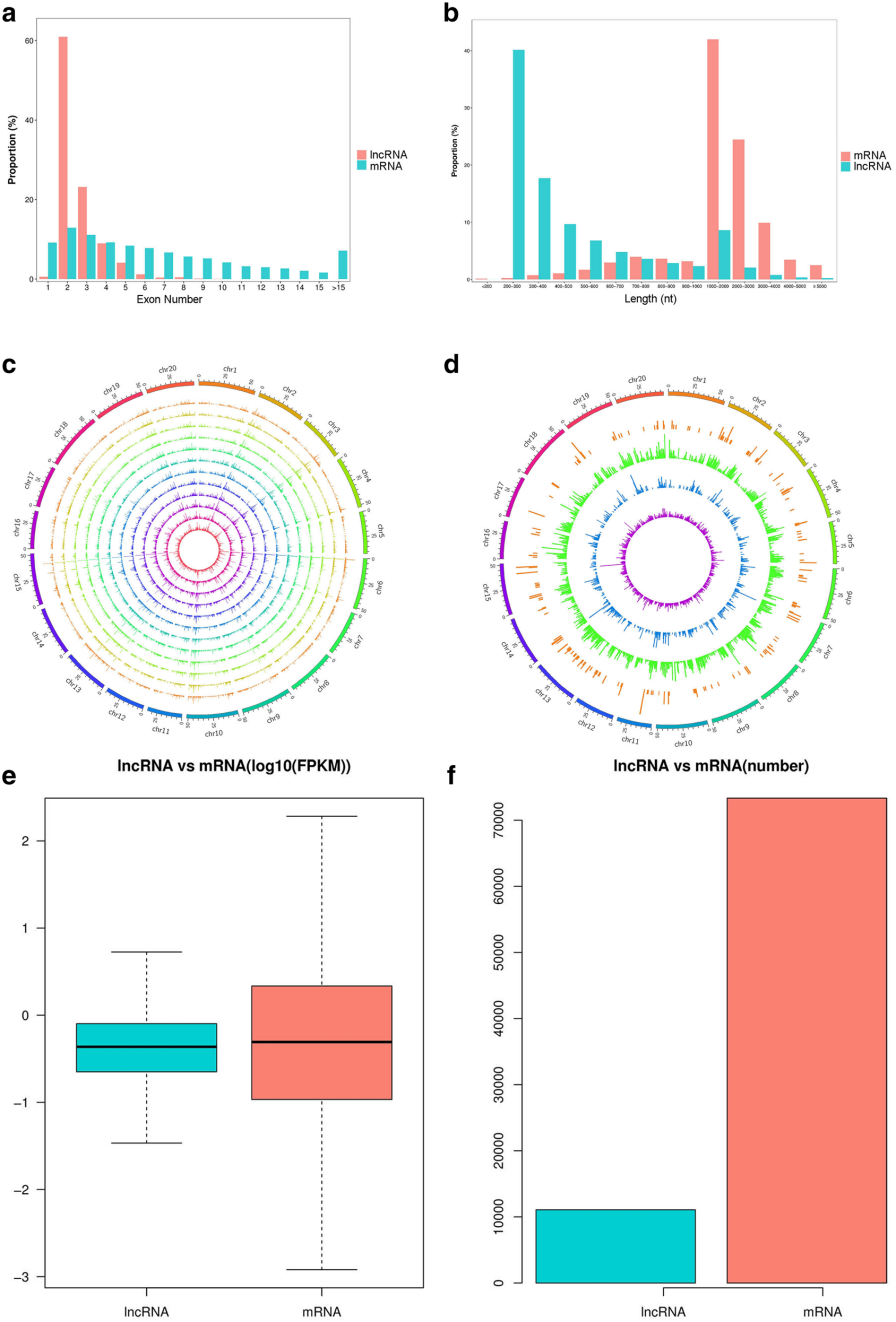
Comparisons of lncRNAs and protein-coding mRNAs in soybean. (**a**) Numbers of exons in lncRNAs and protein-coding mRNAs. (**b**) Comparison of the lengths between lncRNAs and protein-coding mRNAs. (**c**) The expression levels of lncRNAs along the 20 soybean chromosomes. The outermost layer represents all the soybean chromosomes (Chr0120). The inner layer of the figure represents data from three samples per group, which are JN_40, JN_30, MT_40 and MT_30 from the outside to the inside. d. Distribution of different types of lncRNAs. The circles are Antisense, Intronic, Sense and lincRNA from outside to inside. The (**c**) and (**d**) in Fig. 4 are made from the open software circos (v0.63, http://circos.ca/). Expression levels of lncRNAs and mRNAs in samples; (**f**). Numbers of lncRNAs and mRNAs in the samples.

The lncRNA transcripts and gene characteristics identified were analyzed and compared with mRNAs encoded by soybean proteins. First, lncRNAs were analyzed on the basis of the locations of corresponding protein-coding genes. Among them, 5,089 (13.0%) were intronic lncRNAs, 4,015 (10.2%) were long intervening/intergenic noncoding RNAs, 1,221 (3.1%) were sense-lncRNAs and 255 (0.6%) were antisense-lncRNAs (Fig. 4b). Most of the lncRNAs had fewer exons than the protein-coding mRNAs. For example, 937 (61.0%) lncRNAs contained two exons, accounting for the highest proportion. The number of lncRNAs containing more than two exons decreased gradually. The protein-coding genes contained a median of eight exons (Fig. 5a). Additionally, the average length of the lncRNAs was shorter than that of the protein-coding mRNAs (Fig. 5b). In transcripts less than 1 kb, the proportion of lncRNAs was significantly higher than that of mRNAs, while between 1 and 5 kb, the proportion of lncRNAs was lower than that of mRNAs. All the lncRNAs were located on the 20 chromosomes of the soybean genome (Fig. 5c). Different types of lncRNAs were also found in separate chromosomal regions (Fig. 5d). Furthermore, the expression level of each transcript was assessed using the segments per million mapping read segments per kilobase segment model, which indicated that the total expression levels and numbers of lncRNAs were lower than those of mRNAs (Fig. 5e,f).

### Analysis of differentially expressed lncRNAs in soybean

**Figure 6:**
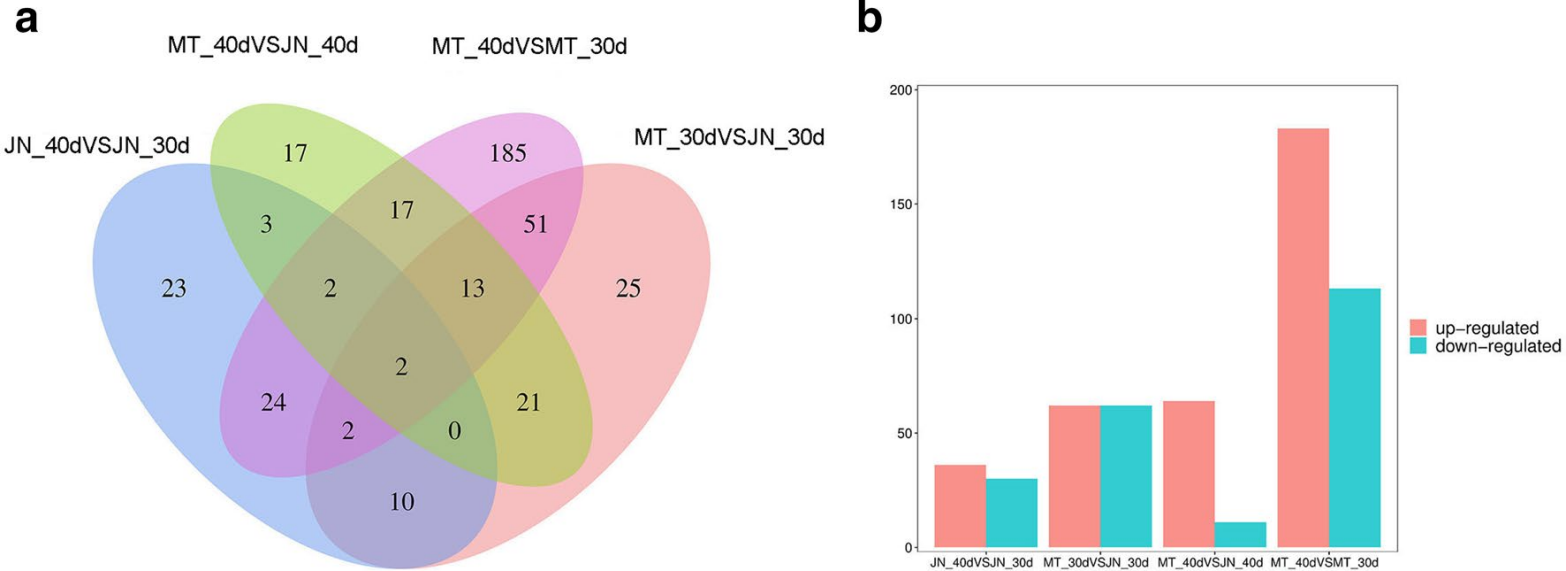
Differentially expressed lncRNAs in soybean. Venn diagrams showing the number of common and specific lncRNAs in comparisons among the four libraries. (**a**) Venn diagram of differentially expressed lncRNAs. (**b**) Regulation of differentially expressed lncRNAs in four different comparisons.

To analyze the expression patterns of lncRNAs from two different soybean materials at 30 and 40 d, the differential expressions of MT_40, MT_30, JN_40 and JN_30 were compared. The criteria log2 (fold change) > 1 or log2 (fold change) < 1 and a statistically significant p value ≤ 0.05 were used for identifying differentially expressed lncRNAs. In total, 561 differentially expressed lncRNAs were identified from four different comparison groups. Among them, 66, 124, 75 and 296 differentially expressed lncRNAs were identified in JN_40 versus JN_30, MT_30 versus JN_30, MT_40 versus JN_40 and MT_40 versus MT_30, respectively (Fig. 6a, Supplementary Table 4). In the MT_40 versus MT30 group, the number of differentially expressed lncRNAs was significantly greater than in the other groups. There were two common differentially expressed lncRNAs in the four comparison groups. Additionally, among the 561 differentially expressed lncRNAs identified in the four groups, 345 were up-regulated and 216 were down-regulated (Fig. 6b). In the MT_30 versus JN_30 comparison group, the numbers of up-regulated and down-regulated lncRNAs were both 62. In MT_40 versus MT30, the number of up-regulated and down-regulated lncRNAs were 183 and 113, respectively.

**Figure 7:**
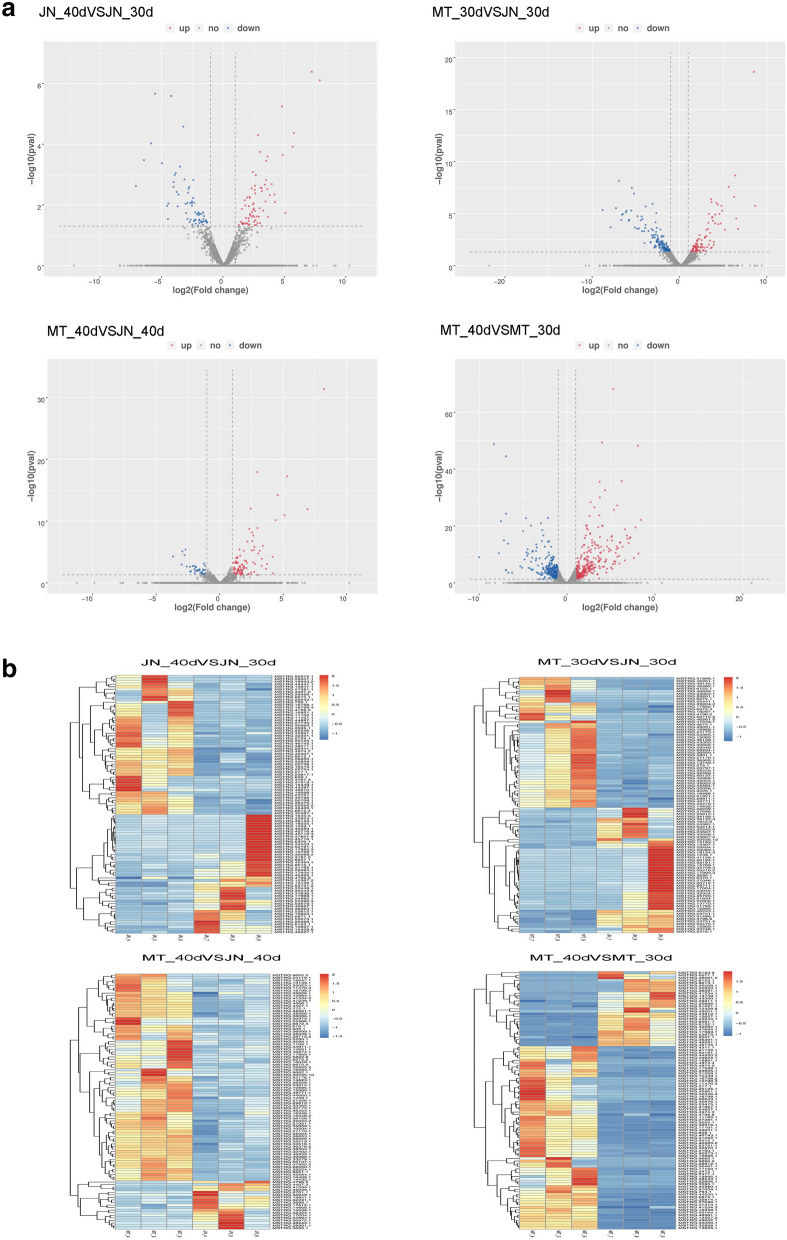
Differentially expressed lncRNAs in soybean. (**a**) Significantly up-regulated (red dots) and down-regulated (blue dots) lncRNAs. (**b**) Clustering of 561 differentially expressed lncRNAs in soybean. The (**b**) in Fig. 6 is made from the open software pheatmap (v1.0.12, https://cran.r-project.org/web/packages/pheatmap/).

To further study the expression patterns of lncRNAs in soybean, a systematic cluster analysis was performed for all 561 differentially expressed lncRNAs (Fig. 7a, b). Most lncRNAs had specific expression patterns in different materials at different stages. Some lncRNAs were differentially expressed in different materials at the same time. For example. MSTRG.29201.1 and MSTRG.4796.5 were up-regulated in MT_40 versus MT_30 but down-regulated in JN_40 versus JN_30. These differentially expressed lncRNAs may have special biological functions in soybean and play important regulatory roles.

### Functional analysis of differentially expressed lncRNAs

**Figure 8:**
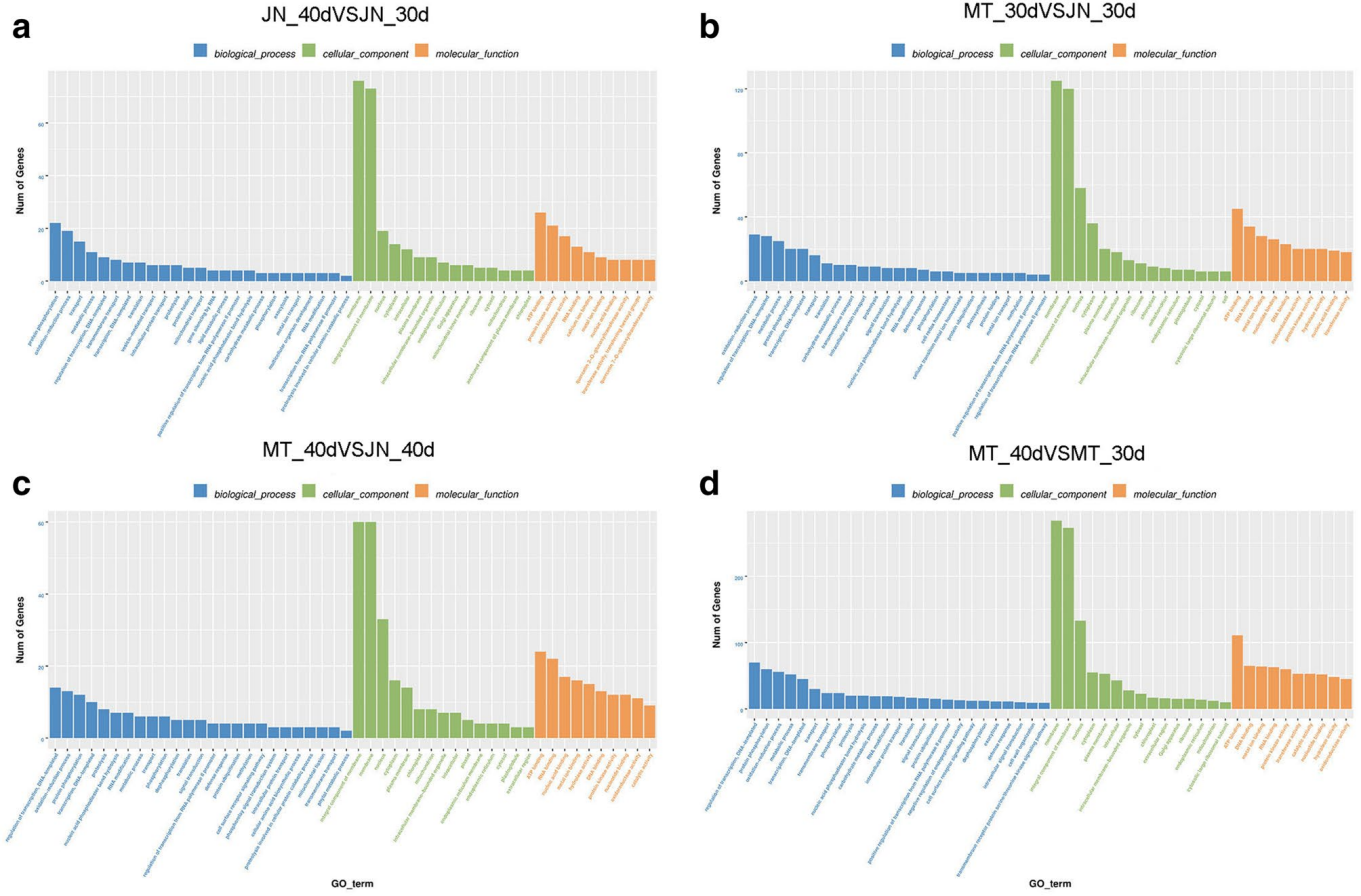
Gene ontology analysis of target protein-coding genes of differentially expressed lncRNAs in soybean. (**a**) JN_40 versus JN_30, (**b**) MT_30 versus JN_30, (**c**) MT_40 versus JN_40, (**b**). MT_40 versus MT_30. MT_40, MT72 at 40 d after flowering, MT_30, MT72 at 30 d, JN_40, JN18 at 40 d, JN_30, JN18 at 30 d.

**Figure 9:**
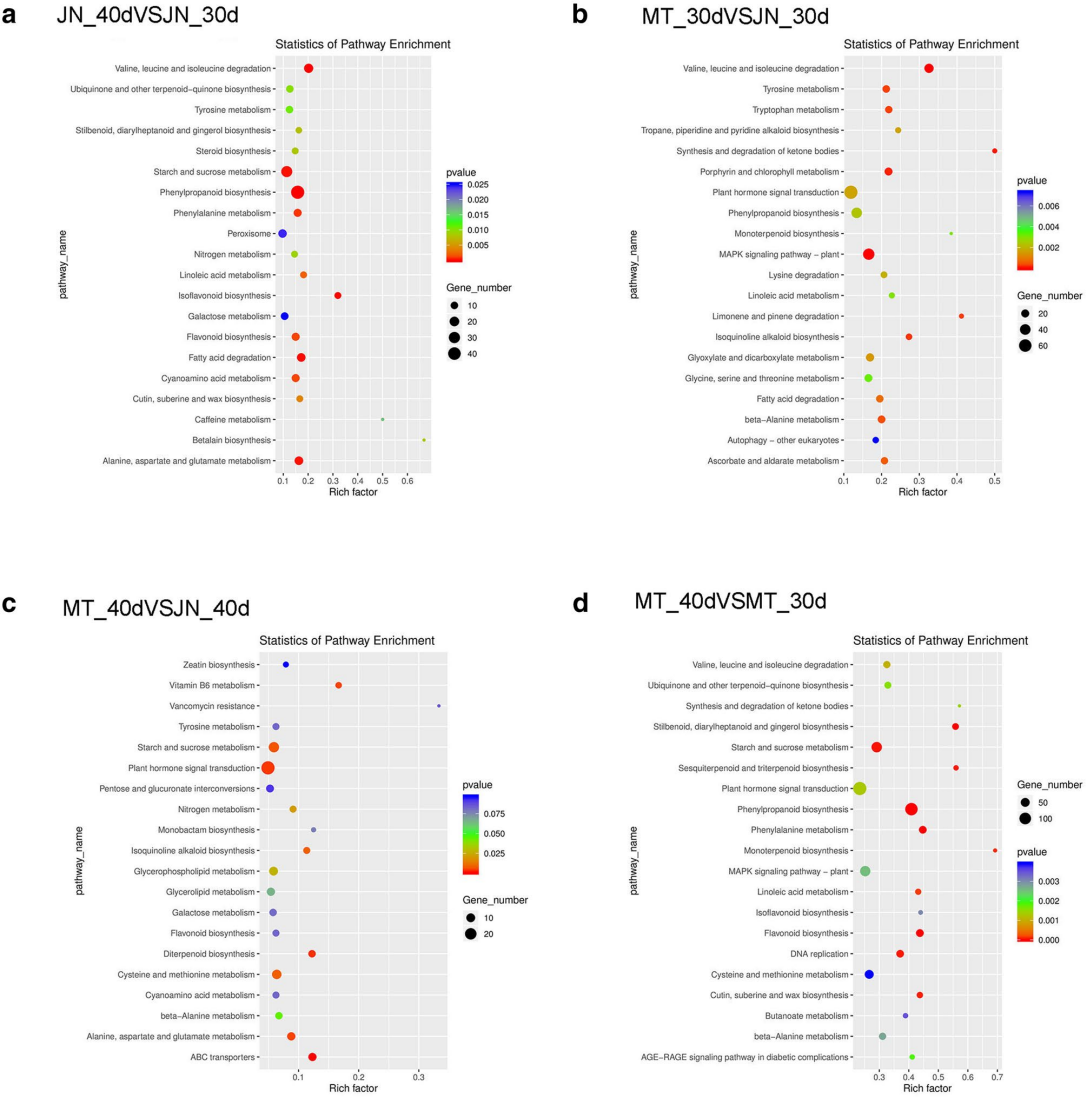
KEGG analysis of target protein-coding genes of differentially expressed lncRNAs in soybean. (**a**) JN_40 versus JN_30, (**b**) MT_30 versus JN_30, (**c**) MT_40 versus JN_40, (**d**) MT_40 versus MT_30. MT_40, MT72 at 40 d after flowering, MT_30, MT72 at 30 d, JN_40, JN18 at 40 d, JN_30, JN18 at 30 d.

To further explore the potential functions of lncRNAs, possible targets of differentially expressed lncRNAs were subjected to a GO analysis. The results showed that some lncRNAs may have fatty acid transport (GO:0,015,908), lipid metabolic process (GO:0,006,629) and other functions related to oil synthesis. Some lncRNAs may also be related to plant-stress responses (GO:0,006,950) and oxidation–reduction processes (GO:0,055,114). Furthermore, several other important biological processes were enriched, such as signal transduction (GO:0,007,165), integral component of membrane (GO:0,016,021) and protein phosphorylation (GO:0,006,468) (Fig. 8a-d). Some genes related to lipid synthesis have been identified as targets of lncRNAs. This suggests that some differentially expressed lncRNAs affect the soybean oil anabolic pathway by regulating the expression of related protein-coding genes. Additionally, the KEGG pathway analysis revealed that these protein-coding genes were significantly enriched in 30 metabolic pathways (Fig. 9a-d), such as fatty acid biosynthesis (KEGG:00,061), fatty acid degradation (KEGG:00,071), and alpha-linolenic acid metabolism (KEGG:00,592), as well as several common metabolic pathways, such as glycolysis/gluconeogenesis (KEGG:00010), pentose phosphate pathway (KEGG:00,030) and flavonoid biosynthesis (KEGG:00,941). Thus, some lncRNAs may have regulatory effects on soybean seed oil synthesis by influencing the fatty acid metabolic pathway.

### Analysis of ceRNAs network reveals the potential functions of lncRNAs in fatty acid synthesis

**Figure 10:**
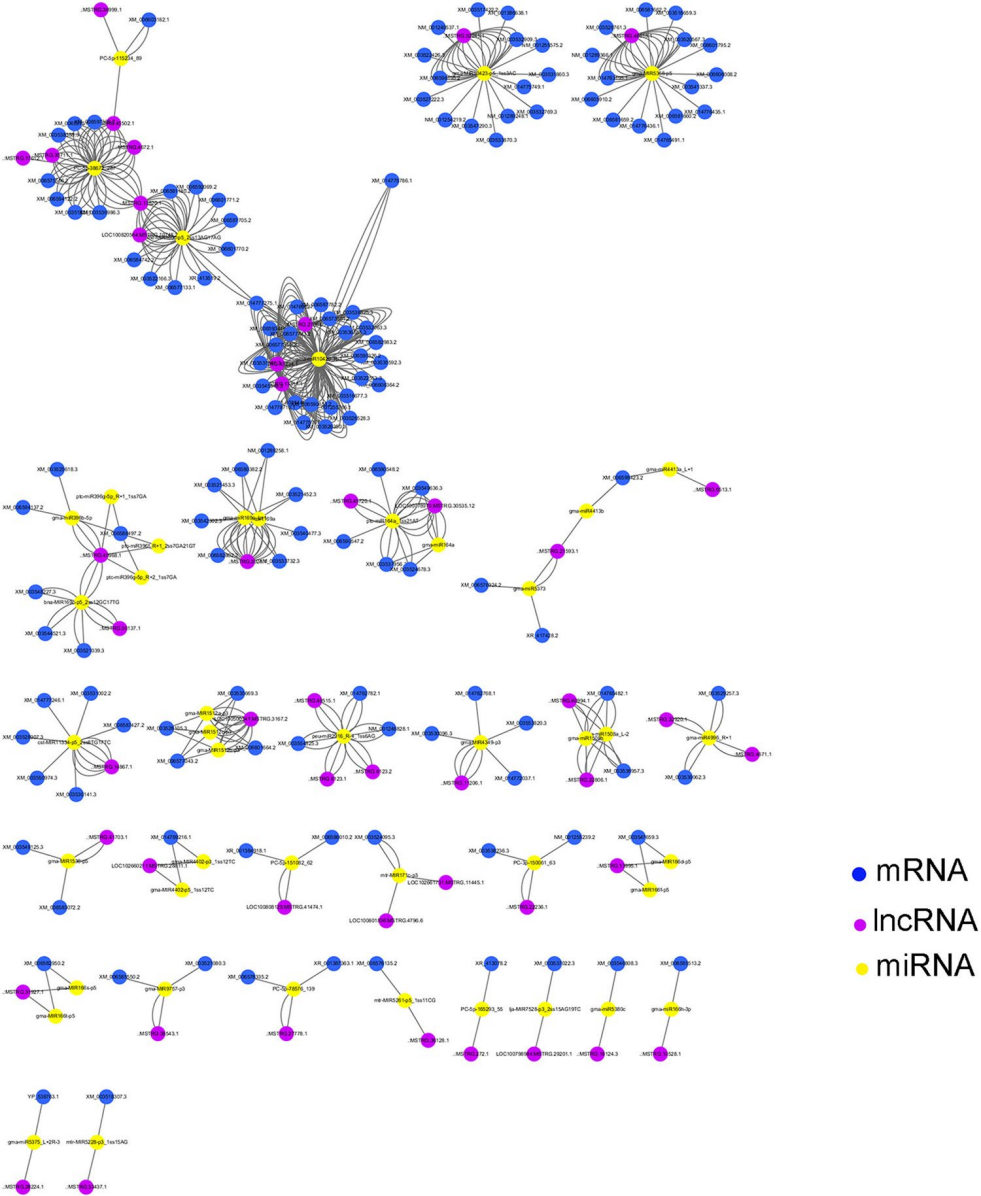
lncRNA-miRNA-mRNA network. The yellow, purple and blue represent miRNAs, lncRNAs and mRNA transcripts, respectively. The Fig. 9 is made from this open software Cytoscape (v3.7.0, http://www.cytoscape.org/).

A total of 209 transcription factors, 65 genes and 10 metabolic pathways related to fatty acid synthesis were identified from transcriptome data. To identify potential lncRNAs related to lipid biosynthesis, we established the co-expression network of lncRNAs-miRNAs-mRNAs predicted to be related to fatty acid biosynthesis. To construct the lncRNAs-miRNAs-mRNAs network, the miRNA expression profiles of ‘MT72′ and ‘JN18′ in soybean at 30 and 40 d after flowering were analyzed. A total of 4,028 miRNAs were found, and 766 miRNAs were differentially expressed. The targets of the 766 differentially expressed miRNA transcripts and lncRNAs were predicted. A network was constructed for each comparison using the identified differentially expressed genes (DEGs). In total, 46 lncRNAs, 46 miRNAs and 137 mRNAs were found to be related (Fig. 10, Supplementary Table 5). GO and KEGG pathway analysis were performed for the target mRNAs of the lncRNAs. A total of 10 lncRNAs may regulate target genes through miRNAs to participate in fatty acid transport processes and regulate fatty acid transporter activities, and thus, they have the potential to influence oil synthesis. For example, the gene XM_003538388.3 has potential functions in lipid metabolic process (GO:0,006,629), including fatty acid synthesis pathways. The possible target genes of the three lncRNAs, MSTRG.35711.1, MSTRG.4672.1, and MSTRG.13820.1 will be all XM_00353838.3. At the same time, these three lncRNAs and miRNAs pc-5p-38672_287 showed spongy mechanisms. Therefore, these three lncRNAs may have functions related to fatty acid synthesis. In addition, the gene XM_003548227.3 has potential functions related to fatty acid transport activity (GO:0,015,245) and fatty acid transport (GO:0,015,908). Because the target gene of lncRNA MSTRG.40968.1 and MSTRG.50137.1 is XM_00354827.3. They also interact with a miRNA BNa-MIR169C-p5_2ss12GC17TG. Therefore, we predict that these two lncRNAs MSTRG.40968.1 and MSTRG.50137.1 may have functions related to fatty acid synthesis. There is also a potential target gene of lncRNA MSTRG.45502.1 with potential functions such as lipid transport (GO:0,006,869), which can indirectly regulate fatty acid transport. Through the analysis of KEGG metabolic pathway, there were 5 metabolic pathways related to fatty acid synthesis. For example, target gene XM_006588497.2 00,061 of lncRNAs MSTRG.40968.1 may be involved in fatty acid biosynthesis (KEGG:00,061) and fatty acid degradation (KEGG:00,071). This gene may be involved in the biosynthesis of long-chain acyl-coenzyme A (CoA) Synthetase (LACS). As an enzyme included in the ACS family, LACS can catalyze the synthesis of fatty acyl coenzyme A, which plays an important role in the anabolism and catabolism of fatty acids. Most importantly, multiple target genes of MSTRG.48618.1 lncRNA, such as NM_001289366.6, may be involved in the pathway alpha-Linolenic acid metabolism (KEGG:00,592). This gene will directly regulate the metabolism of unsaturated fatty acids and is an important gene in subsequent studies. Moreover, the target genes XM_003532063.3 of MSTRG.13311.1, MSTRG.27064.1 and MSTRG.43234.1 may be related to flavonoid biosynthesis (KEGG:00,941). Flavonoids have a certain effect on the accumulation of fatty acids in plants, which will indirectly affect the biosynthesis of unsaturated fatty acids. The results also showed that more than 10 transcription factors in soybean C3H family may regulate target genes and thus regulate fatty acid synthesis. These results will provide a lot of meaningful data for the subsequent studies on the synthesis of soybean fatty acids, especially unsaturated fatty acids (Supplementary Table 6).

### QPCR-based validation of differentially expressed lncRNAs and target genes

**Figure 11:**
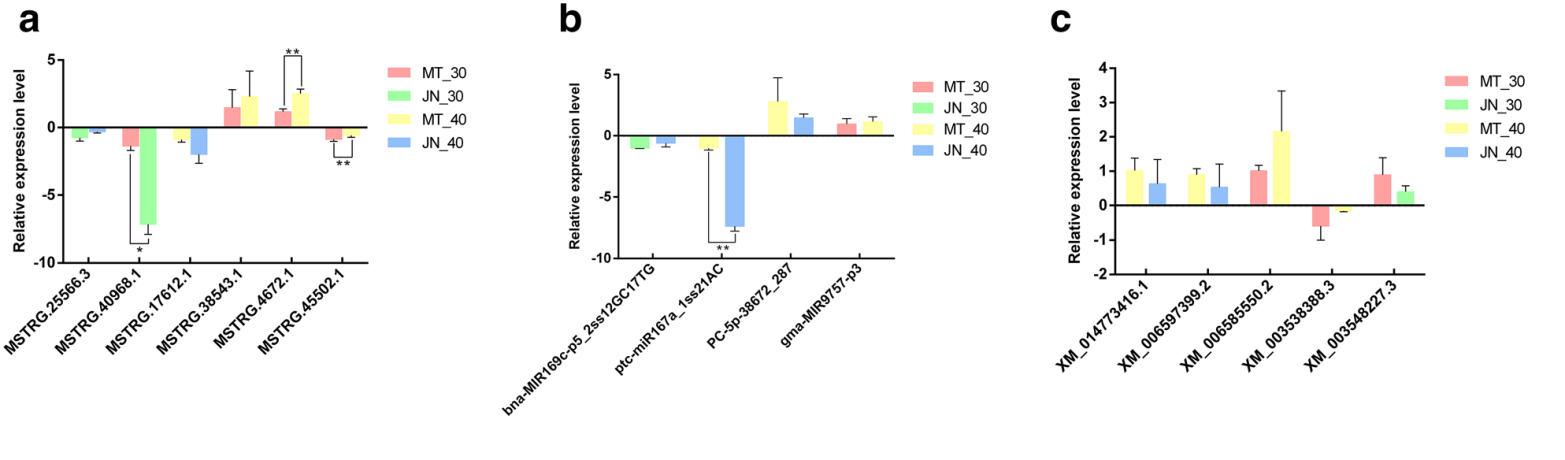
Validation of the expression pattern of four highly expressed lncRNAs by real-time PCR. *represents *p* < 0.05, there is statistical difference, **represents *p* < 0.01 with statistically significant difference.

To verify RNA-seq data, six lncRNAs were randomly selected and their expression verified using qRT-PCR (Fig. 11a-c, Supplementary Table 7). The qRT-PCR results showed that the expression patterns of four lncRNAs were consistent with the high-throughput sequencing results. The lncRNAs MSTRG.25566.3, MSTRG.17612.1 and MSTRG.45502.1 showed down-regulated expression levels in the qRT-PCR results, confirming the RNA-seq results. Similarly, the lncRNA MSTRG.38543.1 was also confirmed to have an up-regulated expression level. These results indicate that the lncRNA expression levels predicted by RNA-seq are highly reliable. To verify the relationships among lncRNAs, miRNAs and their targeted protein-coding genes, the expression levels of four miRNAs and five potential target genes of lncRNAs were verified using qRT-PCR. The expression levels of lncRNA MSTRG.45502.1 and the potential target XM_003538388.3 were down-regulated, while the expression level of the related miRNA PC-5P-38672_287 was up-regulated. This suggested that lncRNA MSTRG.45502.1 is related to the miRNA PC-5P-38672_287 through competitive binding of the target XM_003538388. Additionally, the target XM_003538388 is involved in the regulation of lipid metabolic processes. These findings indicated that some differentially expressed lncRNAs were found related to putative *cis*-acting target genes and predicted to be involved in biological processes related to soybean oil synthesis.

## Discussion

In recent years, lncRNAs have attracted increasing attention in various fields, but research on plant lncRNAs is lagging behind that of animals. However, the regulatory roles of lncRNAs in plants cannot be ignored. At present, their roles have been studied in maize (*Z. mays* L.)^25^, rice (*O. sativa* L.)^26^, Arabidopsis (*A. thaliana* L.)^27^, peanut (*Arachis hypogaea* L.)^28,29^ and other plants ^30–33^. However, the roles of lncRNAs in soybean, especially those related to oil synthesis, have not been reported. Soybean oil synthesis is a complex biological process regulated by the coordinated expression of many key genes. Dynamic changes in the soybean fatty acid content occur during soybean development. Young pods of mutants ‘MT72′ and wild-type ‘JN18′ at 30 and 40 d after flowering were selected for genome-wide analysis of lncRNAs using high-throughput sequencing technology. We identified two common lncRNAs between ‘MT72′ and ‘JN18′ at the different developmental stages. Compared with mRNAs that encode proteins, lncRNAs had shorter sequence lengths, fewer exons and lower expression levels, which was consistent with the results of previous studies on other plants^28,34^. A total of 561 lncRNAs were differentially expressed as determined by analyzing the two materials at different development stages. However, the expression of most lncRNAs was specific to different materials at distinct stages, indicating that lncRNAs have high developmental specificity. It is important to identify and characterize the functions and regulatory mechanisms of these lncRNAs during plant development and stress responses.

The oil content of seed is the most important quality characteristic of soybean, and increasing the soybean oil content is the ultimate goal of most crop breeding programs. With an improved understanding of fatty acid synthesis, and metabolism pathways and key enzyme-encoding genes involved in plant seed oil production, genetic engineering methods may be adopted to regulate the fatty acid metabolic pathways in plant seeds, change the fatty acid composition and improve both the yield and quality of seed oils. In 2018, Yin analyzed the expression profiles of lncRNAs during the seed development of peony varieties with high and low alpha-linolenic acid contents^21^. In total, 2,785 differentially expressed lncRNAs were found. Because lncRNAs could be involved in regulating seed development by modulating the expression of their *cis*-acting target genes. They constructed a co-expression network composed of lncRNAs and target genes, which predicted 39 target genes related to fatty acid and fat biosynthesis.

These target genes may also be involved in signal transduction and multicellular, metabolic and immune system processes, as well as the regulation of these biological processes. This suggests that lncRNAs play key roles in the regulation of gene expression. In this study, the lncRNAs associated with fatty acid transport and lipid synthesis were hypothesized to have potential regulatory roles in soybean oil synthesis. The lncRNAs MSTRG.50137.1 and MSTRG.40968.1 were significantly correlated with the expression patterns of fatty acid transporter genes, and the target mRNAs of lncRNAs MSTRG.4672.1, MSTRG.45502.1 and MSTRG.13820.1 had potential functions in lipid synthesis. These results are consistent with previous reports on the lncRNAs of B. napus. Shen found that the expression patterns of 13 lncRNAs were significantly correlated with the expression patterns of 8 genes related to lipid synthesis^22^. They speculated that lncRNAs co-expressed with lipid-related genes had potential regulatory roles in the biosynthesis and accumulation of B. napus oil.

To further study the regulatory mechanisms of lncRNAs, we constructed a lncRNAs-miRNAs-mRNAs network, which predicted that some lncRNAs regulate the expression of corresponding target genes through miRNAs. A functional analysis of the ceRNAs network indicated that target gene XM_003544521.3 of the lncRNAs MSTRG.50137.1 and MSTRG.40968.1 might be related to phenylpropanoid biosynthesis. Plant phenylpropane compounds regulate abiotic stress-related processes, such as lignin, flavanols and anthocyanin production. The target gene XM_006595284.2 of lncRNA MSTRG.33053.1 may encode an ABC transporter^35–37^. Abscisic acid improves drought resistance by balancing production, catabolism and transportation in peanut leaves. Furthermore, the target gene XM_014778710.1 of lncRNA MSTRG.43234.1 encodes the zinc-finger protein NFXL1, and the associated mRNA regulates zinc-finger protein production, which improves oil synthesis. These target genes are related to lipid synthesis and abiotic stress responses^29^.

The lncRNAs related to oil synthesis were identified and their regulatory network was characterized. This further enhanced our understanding of the regulatory mechanism of oil synthesis, at a molecular level, in soybean and other oil crops. This is the first report on the expression profiles of lncRNAs involved in soybean oil synthesis. A total of 39,324 lncRNAs were found, and 561 were differentially expressed during the development of mutants and wild-type pods. A functional analysis of these lncRNAs and related protein-coding genes indicated that the former play important roles in soybean oil synthesis. The construction of the lncRNAs-miRNAs-mRNAs network indicated that some lncRNAs regulate the expression of corresponding protein-coding genes through miRNAs interactions. Our results provide new insights into the functions of lncRNAs in soybeans and their expression patterns during soybean seed development. They also provide valuable theoretical and practical bases for cultivating soybean varieties with high oil-synthesis capabilities.

## Materials and methods

### Plant materials

**Figure 12:**
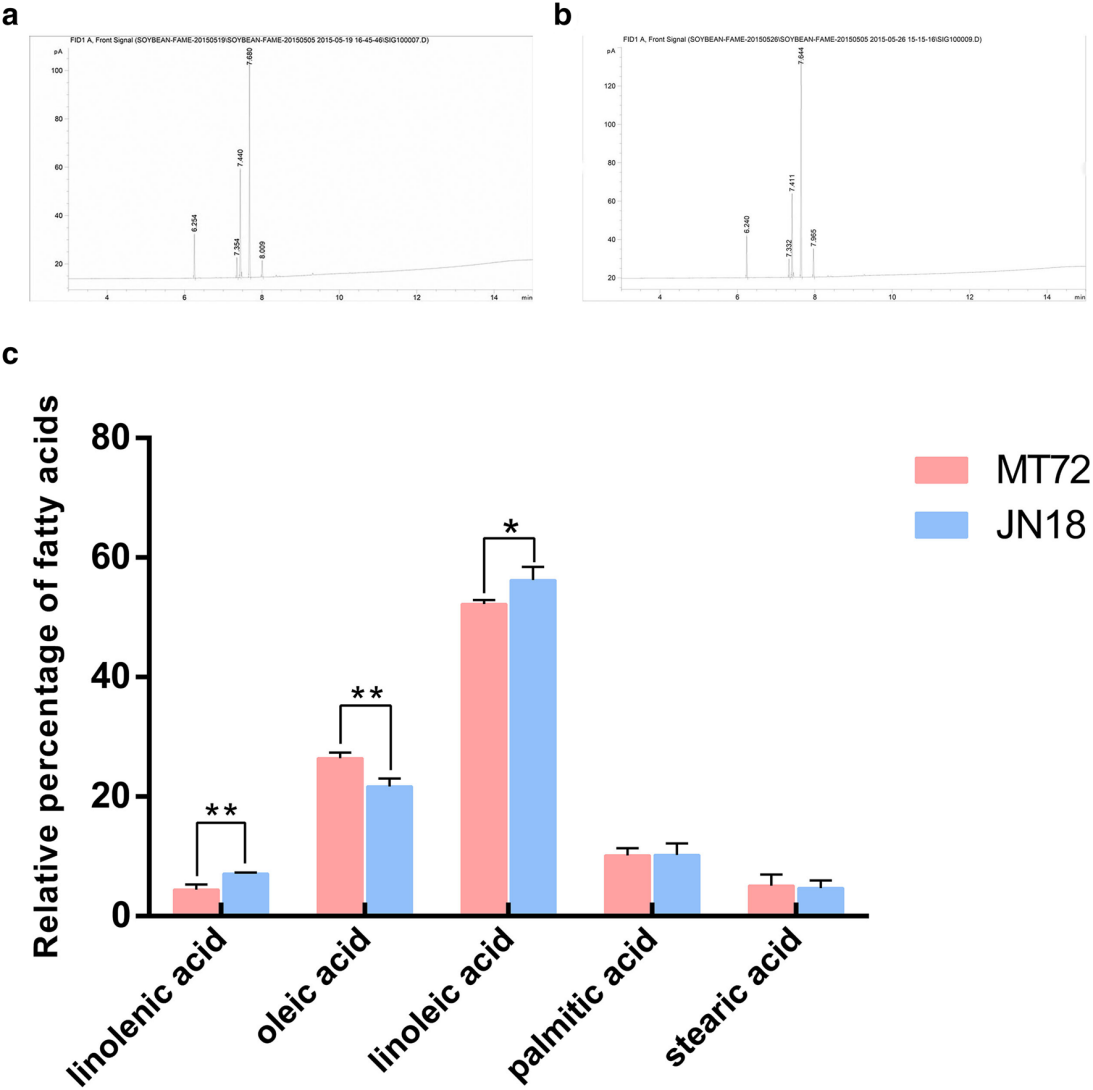
Quality analysis of main fatty acid components of ‘MT72′ and ‘JN18′. (**a**) Gas chromatogram of mutant ‘MT72′. (**b**) Gas chromatogram of mutant ‘JN18′. (The peak times are palmitic acid, stearic acid, oleic acid, linoleic acid and linolenic acid from left to right). (**c**) Bar diagram of ‘MT72′ and ‘JN18′ different fatty acid content. *indicates a statistical difference at *p* < 0.05, ** indicates a statistical difference at *p* < 0.01.

**Figure 13:**
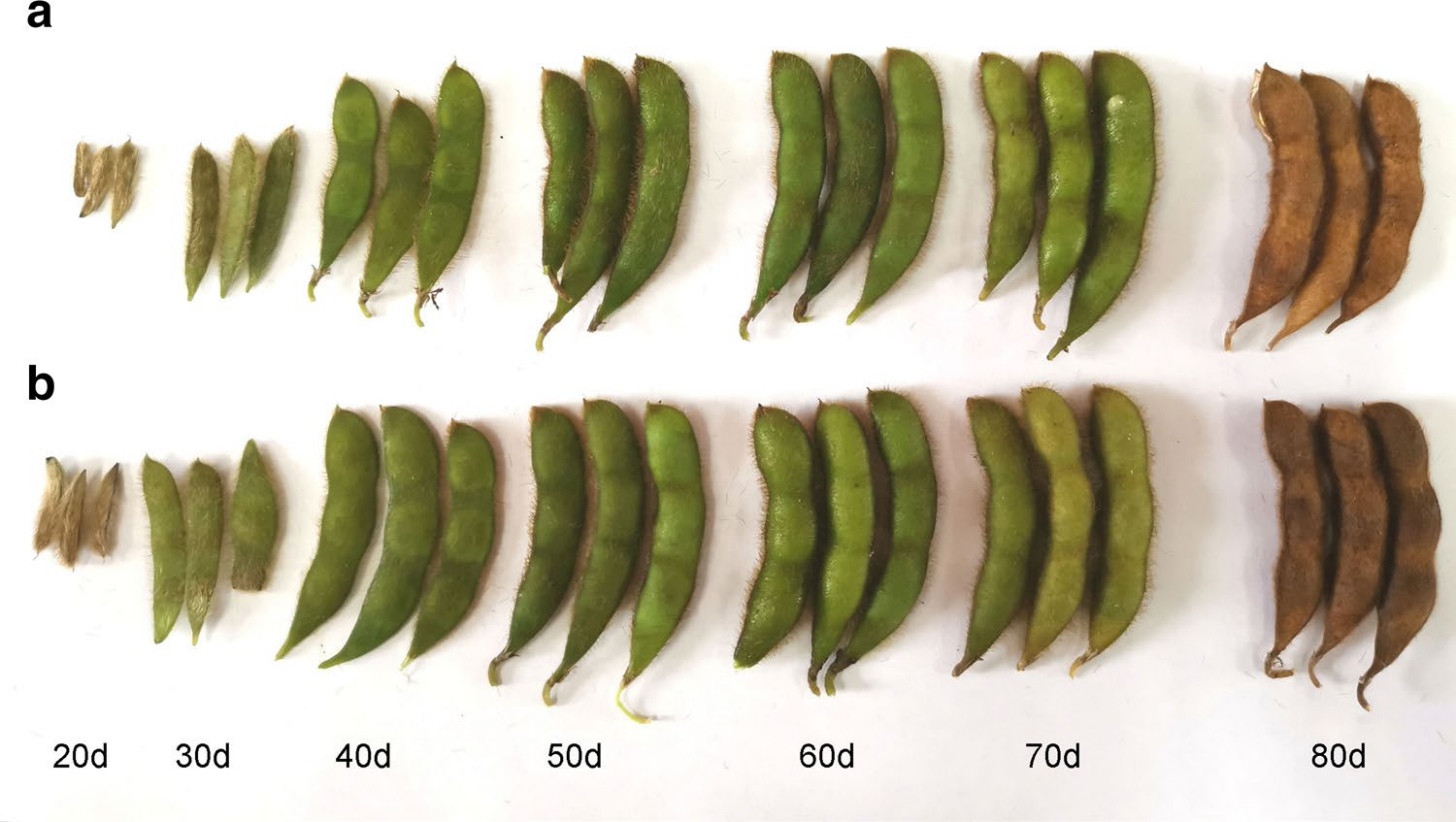
Soybean [*Glycine max* (L.) Merr] materials used in this study. samples were taken seven times from 20 to 80 d after soybean flowering. (**a**) Low-linolenic acid mutant ‘MT72′, (**b**) Control material ‘JN18’.

In 2015, the low-linolenic acid mutant ‘MT72′ was obtained after soybean was exposed to 0.5% ethyl methanesulfonate for 6 h. The results of gas chromatography showed that the unsaturated fatty acid level of ‘MT72′ was 4.41% linolenic acid, 26.46% oleic acid, and 52.22% linoleic acid. And the saturated fatty acid level of ‘MT72′ was 10.20% palmitic acid and 5.11% stearic acid (GC-7890, Agilent, Santa Clara, CA, USA). The high-oil soybean variety ‘JN18′ was determined to contain 7.11% linolenic acid, 21.73% oleic acid, 56.21% linoleic acid, 10.25% palmitic acid and 4.70% stearic acid using gas chromatography (Agilent) at the Biotechnology Center of Jilin Agricultural University in 2006. The content of linolenic acid and oleic acid in ‘MT72′ and ‘JN18′ is significantly different (Fig. 12). In this study, the fourth-generation low-linolenic acid mutant ‘MT72′ was used as the experimental material and ‘JN18′ as the control material. Two groups of soybean pod samples were collected from the experimental and control groups every 10 days (d) starting from 12 d after flowering. Seven samples were taken from the teaching and research base of Jilin Agricultural University from 20 to 80 d after flowering in 2018 (Fig. 13). The pods were wrapped in aluminum foil, quickly frozen in liquid nitrogen and then stored at -80.

### LncRNAs library construction and RNA sequencing

Total RNA from young soybean pods of ‘MT72′ and ‘JN18′ at 30 and 40 d after flowering were extracted using Trizol Reagent (Invitrogen, Carlsbad, CA, USA). Total RNA from each sample was used to prepare RNA-seq libraries in accordance with the protocol of the Ribo-Zero rRNA Removal Kit (Illumina, San Diego, CA, USA), in which ribosomal RNA was depleted from approximately 10 μg of total RNA representing a specific adipose type. The remaining RNAs was fragmented into small pieces using divalent cations at a high temperature. Then, cleaved RNA fragments were reverse-transcribed to create the final cDNA library in accordance with the protocol for the TruSeq Stranded Total RNA Library Prep Kit (Illumina). The lncRNA libraries were sequenced on an Illumina Novaseq 6000 platform (LC Sceiences, Hangzhou, China) with 2 × 150-bp paired-end reads.

### Identification of lncRNAs and differential expression analysis

Firstly, Cutadapt was used to remove the reads that contained adaptor contamination, low quality bases and undetermined bases^23^. Next, the sequence quality was verified using FastQC (http://www.bioinformatics.babraham.ac.uk/projects/fastqc/). We then used HISAT2^38^ to map them to the reference genomes of two soybean varieties. To construct transcriptome, the mapped reads were assembled using StringTie^24^. After final transcriptome generation, StringTie^24^ was used to estimate the expression levels of all the transcripts. Among the remaining transcripts, those with length longer than 200 bp were selected for the protein-coding-score test to calculating the Coding Potential Calculator (CPC)^39^ and Coding-Non-Coding Index (CNCI)^40^. The differentially expressed lncRNAs were selected that had |log2 (fold change) |> 1 and a statistically significant p value ≤ 0.05 using R package’s edgeR^41^.

### Functional annotation and lncRNAs-miRNA-mRNAs interaction network construction

To explore the functions of lncRNAs in soybean development, we predicted the *cis*-target genes of lncRNAs. The enrichment of predicted genes in cellular component, molecular function, and biological process was analyzed using the Gene Ontology (GO) database (http://www.geneontology.org). The pathway analysis of potentially differently expressed genes used the Kyoto Encyclopedia of Genes and Genomes (KEGG) database (http://www.genome.jp/kegg/)^42^. The lncRNAs-miRNAs-mRNAs network was constructed using Cytoscape (version 3.7.0)^43^.

### Quantitative real-time PCR validation

To verify the sequencing results, qRT-PCR was performed to detect the expression levels of lncRNAs, miRNAs and target genes in soybean pods of different groups. Total RNAs of young soybean pods in four stages were extracted using the above methods. They were reverse-transcribed using the UEIris II RT-PCR System with dsDNase (Code R2028, US Everbright Inc., Suzhou, China). Fast Super EvaGreen qPCR Master Mix (Code S2008, US Everbright Inc., Suzhou, China) was used for the qRT-PCR. The qRT-PCR conditions were as follows, initial 3-min denaturation at 95 °C, followed by 40 cycles of 30 s denaturation at 95 °C, 30 s at an appropriate annealing temperature and 30 s extension at 72 °C, followed by a 10 min final extension at 72 °C. All the reactions were performed with three replicates. The relative gene expression values were calculated using the 2^−ΔΔCT^ method^44^.

## Supplementary Information

Supplementary Information

## Data Availability

All data pertaining to the present study has been included in the Figures/supplementary files of the manuscript. The RNA-seq datasets are avail-able in the GEO database (Accession ID: GSE161260, https://www.ncbi.nlm.nih.gov/geo/query/acc.cgi?acc=GSE161260).
